# The Times of Our Lives: Interaction Among Different Biological Periodicities

**DOI:** 10.3389/fnint.2018.00010

**Published:** 2018-03-13

**Authors:** Rodrigo Laje, Patricia V. Agostino, Diego A. Golombek

**Affiliations:** Departamento de Ciencia y Tecnología, Universidad Nacional de Quilmes (UNQ), CONICET, Buenos Aires, Argentina

**Keywords:** biological timing, circadian system, infradian rhythms, ultradian rhythms, interaction, mathematical model

## Abstract

Environmental cycles on Earth display different periodicities, including daily, tidal or annual time scales. Virtually all living organisms have developed temporal mechanisms to adapt to such changes in environmental conditions. These biological timing structures—ranging from microsecond to seasonal timing—may have intrinsic properties and even different clock machinery. However, interaction among these temporal systems may present evolutionary advantages, for example, when species are exposed to changing climatic conditions or different geographic locations. Here, we present and discuss a model that accounts for the circadian regulation of both ultradian (less than 24-h) and infradian (more than 24-h) cycles and for the interaction among the three time scales. We show two clear examples of such interaction: (i) between the circadian clock and the seasonal regulation of the Hypothalamic-Pituitary-Thyroid (HPT) axis; and (ii) between the circadian clock and the hypothalamic-nigrostriatal (HNS) ultradian modulation. This remarkable interplay among the otherwise considered isolated rhythms has been demonstrated to exist in diverse organisms, suggesting an adaptive advantage of multiple scales of biological timing.

## Biological Timing

Biological mechanisms that account for temporal information are ubiquitous and essential for physiology and behavior. Indeed, biological timing comprises distinct time-related processes that span several orders of magnitude, from microsecond to seasonal events (Buhusi and Meck, 2005; Golombek et al., 2014). Among these temporal orders, almost all living organisms are subjected to the influence of the Earth’s rotational cycle of 24 h. This rhythmic pattern, with a period close to 24-h, is called circadian rhythm (from the Latin words *circa dies*, around a day).

It is puzzling that in most cases, different biological time scales have been researched and considered not only independently but also as isolated compartments, with the notable exception of the circadian reading needed for photoperiodic regulation (e.g., Pittendrigh and Daan, 1976). In this perspective we propose a general framework for the interaction of multiple time scales using well-known examples of different periodicities. We also show how modeling experimental manipulations in one timescale are expected to have effects at a different time range. Although several of the feedback handles among clocks and temporal systems are not well understood, we do urge to consider multiple temporalities and their interactions when analyzing a certain physiological or biochemical variable.

### Circadian Timing

Circadian rhythms are the result of self-sustaining and cell-autonomous oscillations generated by circadian clocks. These circadian clocks are characterized by their persistence under constant conditions (endogenous oscillation), temperature-compensation (Q_10_ close to 1), and their entrainment to external input (Dunlap et al., 2004). In mammals, the circadian system presents a hierarchical organization, with the main or central oscillator located in the suprachiasmatic nucleus (SCN) of the hypothalamus. In turn, the SCN coordinates other brain and peripheral oscillators of the rest of the body, such as the paraventricular nucleus (PVN; Abe et al., 2002) and the liver (Stokkan et al., 2001), through endocrine or neural mechanisms. At the molecular level, mammalian cell-autonomous circadian clocks are generated by transcription-translational feedback loops, in which the expression of Period (Per) and Cryptochrome (Cry) genes is suppressed by their protein products (Buhr and Takahashi, 2013). Posttranslational episodes that modulate protein half-life and subcellular localization contribute to these oscillations (Hastings et al., 2014). In addition, a second feedback loop adds robustness to the system, and new actors are still being incorporated in this general molecular scheme. The light/dark cycle is the main synchronizer (also called *Zeitgeber*) of circadian rhythms in mammals (Golombek and Rosenstein, 2010).

## The Circadian Clock as a Modulator of Infradian and Ultradian Rhythms

**Figure 1:**
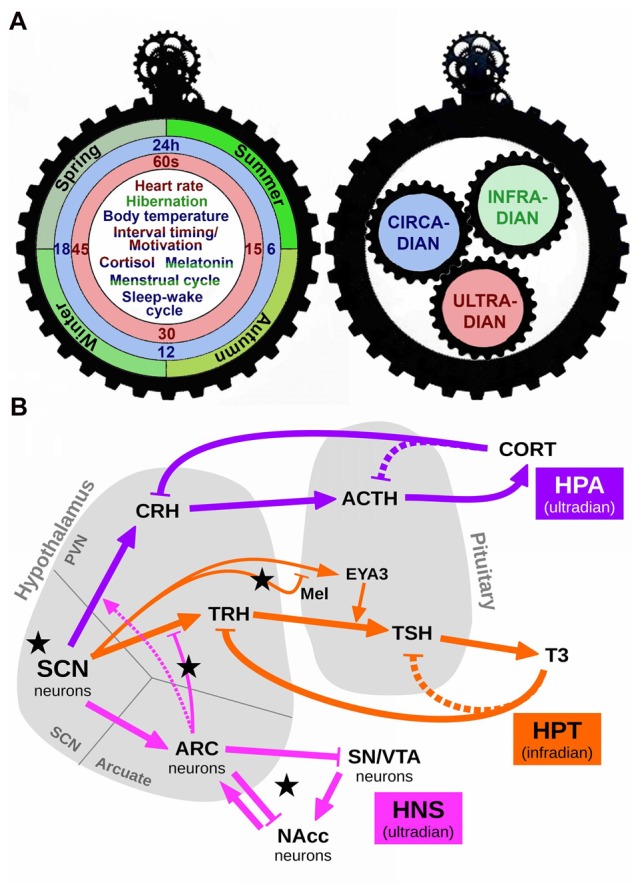
Schematic representation of the interaction among different scales of biological timing. **(A)** The left panel depicts one side of a gear clock containing examples of ultradian (red), circadian (blue) and infradian (green) events. Some events, such as cortisol release or the menstrual cycle, are regulated by more than one scale of biological timing. Thus, cortisol release has both ultradian and circadian components (red/blue), whereas the menstrual cycle presents circadian and infradian regulation (blue/green). The right panel shows the other side of this clock, in which each temporal scale may act as a gear, interacting with the others to produce a fine regulation of temporal events. **(B)** A simplified conceptual model of the various biological rhythms and their interactions. Arrows represent excitation/activation; flat-ended lines represent inhibition/repression. Dashed lines represent known but disregarded interactions. Black stars mark our proposed experimental manipulations: short and long photoperiod (short photoperiod, SP an longphotoperiod, LP, respectively) conveyed by Mel; short Zeitgeber period (SH); fasting, represented by an increased efficacy of the connection ARC→TRH (via higher NPY); slower HNS oscillations due to increased Dopamine (DA). Abbreviations: SCN, suprachiasmatic nucleus; HPA, Hypothalamic-Pituitary-Adrenal axis; HPT, Hypothalamic-Pituitary-Thyroid axis; HNS, Hypothalamic-Nigro-Striatal axis; CRH, corticotropin-releasing hormone; ACTH, adrenocorticotropic hormone; CORT, cortisol/corticosterone; TRH, thyrotropin-releasing hormone; TSH, thyroid-stimulating hormone; T3, triiodothyronine; Mel, melatonin; EYA3, eyes-absent transcriptional coactivator and phosphatase 3; ARC, arcuate nucleus; SN/VTA, substantia nigra/ventral tegmental area; NAcc, nucleus accumbens.

Circadian rhythms are also under the influence of the Earth’s axial tilt, which causes seasonal changes in the length of the day (i.e., photoperiod). By convention, those cyclic events whose period is longer than 24-h are called infradian rhythms, and range from several days (such as the menstrual cycle) to annual cycles (such as hibernation, see Figure 1A). Some examples of infradian rhythms in mammals include migration, hibernation, moulting and pelage growth, and reproductive behavior, all of them synchronized by internal timing mechanisms (Goldman, 2001). Photoperiod is encoded in multiple rhythms driven by the SCN, including electrical activity, gene expression and gating of sensitivity to input stimuli (Coomans et al., 2015). The hormone melatonin (Mel), released by the pineal gland, also provides an endocrine representation of seasonal information (i.e., changes in photoperiod) in many species via the SCN-pineal pathway. How daylength is encoded within the neuronal network of the SCN has been described by experimental and theoretical works (e.g., Myung et al., 2015). We will now present examples of interactions between the circadian system and infradian/ultradian rhythms.

Circadian influence in seasonal hormone release is evident in the circa-monthly rhythm of the Hypothalamic-Pituitary-Gonadal axis and its luteinizing hormone secretion (not included in this model for the sake of extension) and also in the Hypothalamic-Pituitary-Thyroid (HPT) axis. The circadian oscillation of thyroid-releasing hormone (TRH), thyroid-stimulating hormone (TSH), thyroxine (T4) and triiodothyronine (T3) have been well documented (Philippe and Dibner, 2015). In mammals, the infradian regulation of thyroid hormones is mediated via specialized cells (called thyrotrophs) located in the pars tuberalis (PT) of the pituitary gland. According to a current external-coincidence model, the rhythmic Mel signal generates a local circadian oscillation in the PT via induction of the clock gene Cry1 and the circadian-controlled transcriptional co-activator Eyes Absent 3 (EYA3). Independently of day-length, the peak of EYA3 is around 12-h after dark and Mel onset. On short photoperiods (SP, winter season), its peak time falls at night when the Mel level is high, which suppresses EYA3 expression. On long photoperiods (LP, summer season), the phase of EYA3 is now coincident with the daytime, when the Mel level is minimal, and thus EYA3 can be expressed. This leads to coactivation of the TSHb promoter in the PT thyrotoph, driving seasonal deiodinase enzyme (DIO) signaling and TH metabolism (Dupré et al., 2010; Wood et al., 2015; Figure 1B).

Among ultradian rhythms—those oscillations that display a period shorter than 24-h—that are regulated by the circadian system, important examples include the Hypothalamic-Pituitary-Adrenal (HPA) axis and the Hypothalamic-NigroStriatal (referred as HNS in the present text) axis. The HPA axis is the main neuroendocrine system responsible for the response to stress, although also regulates energy balance, emotions and immune system. The SCN generates the circadian regulation via an inhibitory input to the neurons containing corticotropin-releasing hormone (CRH) in the PVN. These neurons in turn release CRH, which is transported through axonal projections to the median eminence (ME) where it is secreted into the hypothalamic-pituitary portal blood system to stimulate the release of adrenocorticotropic hormone (ACTH) from corticotroph cells in the anterior pituitary into the systemic circulation. This leads to increased secretion of glucocorticoids (cortisol in humans and corticosterone in rodents—both referred as CORT the present manuscript) from adrenal cortex, anticipating the activity phase (Lightman, 2016; Figure 1B). Thus, the HPA axis exhibits both circadian and ultradian rhythms in CORT release. These variations are largely related to the sleep/wake cycle (Postnova et al., 2013). In the HNS axis, the arcuate nucleus (ARC) of the hypothalamus generates neuroendocrine ultradian rhythms closely related to feeding and locomotor behavior (Prendergast and Zucker, 2016). Also, there is a circadian-dependent inhibitory influence of the SCN on the neuronal activation in the ARC to hypoglycemia, indicating a gating role of the SCN for metabolic information reaching the brain via the ARC. The ARC in turn influences the activity of the SCN, indicating a reciprocal connection between these nuclei (Buijs et al., 2017). The ARC is also connected to the striatum, the substantia nigra (SN) and the ventral tegmental area (VTA). There is a dopamine (DA)-sensitive ultradian oscillator in the striatum, potently modulated by SN/VTA. Indeed, it has been hypothesized that these discrete structures may interact in generating ultradian rhythms via a network comprised of the SCN, ARC and the SN/VTA signaling to the nucleus accumbens (NAc; Prendergast and Zucker, 2016).

We have recently provided evidence of a circadian modulation of a specific mechanism of higher frequency, corresponding to seconds-to-minutes interval time estimation in rodents. Indeed, interval timing varies throughout the day under entrained (light-dark) or free-running (constant dark) conditions, and is completely blunted under functional circadian arrhythmicity conditions induced by constant light (Agostino et al., 2011). This circadian variation appears to be based upon modulation of the underlying dopaminergic (DA) mechanism, both at the neurotransmitter and receptor levels (Bussi et al., 2014; unpublished results). It remains to be established whether this circadian-ultradian monoaminergic interplay is also of importance in other DA-related functions, including its role in neuroendocrine and behavioral regulation. It also remains to be elucidated why, despite several works that support an interaction between the circadian system and interval timing (reviewed in Golombek et al., 2014; see also Balzani et al., 2016), there is discrepancy in the literature about the effect of circadian disruption on temporal processing (e.g., Lewis et al., 2003; Cordes and Gallistel, 2008; Petersen and Mistlberger, 2017).

## Conceptual Model, Mathematical Implementation and Predictions

In order to analyze the interplay among the different time scales described above, we propose a coarse-grained conceptual model of some of the various infradian, circadian and ultradian rhythm generators and their interactions (Figure 1B). The biochemical species and arrows in this model were chosen to depict a simplified yet well-supported representation of reality. We translated this conceptual model into a mathematical implementation intended to qualitatively reproduce the temporal evolution of known components of the clocks’ machinery, with a level of description intermediate between abstract models (low number of variables and parameters but difficult to associate to real quantities) and detailed molecular models (easy interpretation but high number of variables and parameters). A detailed description of the mathematical model, our assumptions and simplifications, exemplary time series and MATLAB code can be found in the Supplementary Material. Some noticeable simplifications are worth considering here and include, for instance, dropping the long-established negative feedback from CORT to ACTH, and from T3 to TSH (both represented by dashed lines in Figure 1B), in order to have a simpler mathematical description while keeping a nonlinear oscillating system (our choice was Goodwin’s well-known molecular mathematical model; Goodwin, 1965; Gonze, 2011). A second important simplification is that our model reproduces a seasonal thyroid profile with higher T3 during summer as observed in seasonally breeding species (Yoshimura et al., 2003; Hanon et al., 2008; for a review see Dardente et al., 2014), while in other species, including humans, lower T3 values occur during summer instead (Maes et al., 1997; Smals et al., 1997). In addition, some medical and external conditions make TSH values vary without corresponding changes in T3 due to various feedback paths not considered here (see for instance Pappa and Alevizaki, 2013). In any case, a higher T3 in our model is to be interpreted as an HPT axis with increased overall activity.

**Figure 2:**
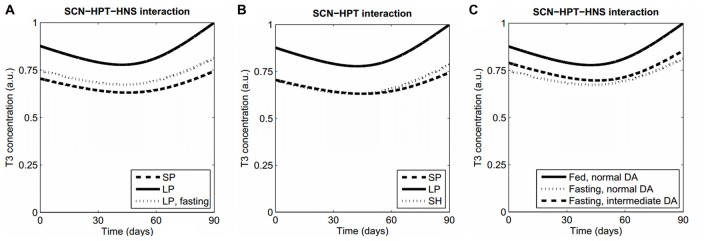
Model features and predictions. **(A)** Our model qualitatively reproduces the lowering of T3 levels both in a SP and during fasting, as compared with LP levels. **(B)** Prediction 1. Keeping a LP in an artificially short Zeitgeber period (SH) will produce a winter T3 profile as in normal Zeitgeber period with a SP. **(C)** When fasting, higher concentrations of striatal DA will lead to normal T3 values. All simulations were performed with the same set of parameter values, except for those parameters that vary because of the proposed manipulations (Supplementary Table S1, Data Sheet 2).

In order to show the behavior of our model, we first simulated the following “normal” conditions: (a) short-photoperiod (SP); (b) long-photoperiod (LP); and (c) fasting (represented by an increased efficacy of ARC to inhibit TRH production due to high NPY; Sarkar and Lechan, 2003). Figure 2A shows the resulting time series of T3. Both the SP and fasting conditions lead to decreased levels of T3 in comparison with LP, as expected (seasonal T3: Dardente et al., 2014; fasting: Harvey and Klandorf, 1983; Sarkar and Lechan, 2003). See Supplementary Figure S1 for the short-term evolution of ultradian oscillations.

A most interesting feature of this mathematical implementation is that a number of predictions about unobserved behaviors can be drawn from it, in particular regarding interactions between different time scales:

a. *Interaction between circadian and infradian rhythms: a winter thyroid profile will be recovered at LP if the Zeitgeber has a sufficiently short cycle*. The observation that EYA3 has a peak at a fixed time after dark onset (Dupré et al., 2010; Wood et al., 2015) led us to consider that artificially shortening the Zeitgeber period while keeping a LP would make the Mel and EYA3 peaks to coincide as in a regular winter Zeitgeber. This would suppress the expression of EYA3, which in turn would prevent the activation of the TSHb promoter and finally produce a low activation of the HPT axis. Numerical simulations supporting this prediction are shown in Figure 2B and Supplementary Figure S2.
b. *Interaction between ultradian and infradian rhythms: increasing the concentration of extracellular DA in the striatum will produce higher T3 profiles when fasting*. The normal inhibition of TRH expression (HPT axis) due to ARC neuronal activity (HNS axis) depends on HNS-SCN synchronization. It is known that increased levels of striatal DA lengthen the period of ultradian oscillations from about 4 h to 20 h and probably more (Blum et al., 2014), which induces desynchrony between the HNS and the SCN. Thus we hypothesized that modeling an experimental manipulation of DA levels leading to HNS-SCN desynchronization might cause abnormal thyroid profiles, and this would occur only when the HPT axis is more sensitive to ARC activity like during fasting (high NPY levels). Numerical simulations in Figure 2C reflect this prediction; similar results are obtained either at long or SPs.

## Conclusion

In summary, we propose a putative neural network that may participate in the interconnection of different scales of biological timing in order to finely regulate behavioral and physiological rhythms. We can certainly consider two sides of biological timing. On the one hand, we tend to identify and study different temporal phenotypes in the organism as a whole (including behavioral, molecular and physiological parameters), thus considering it a constant interplay between the environment and the chronological responses of the body (Figure 1A, left panel). But when we turn the clock upside down, we usually isolate different oscillatory mechanisms in order to understand them in detail, not necessarily considering the essential interaction between the convergent sizes and functions of biological gears (Figure 1A, right panel). A unified view of biological timing and the interaction among its various constituents and time scales can help understand conflicting or contradictory results. Considering the model presented in this perspective, for instance, we predict that the effect of high striatal DA is an increased HPT activity (ultradian-infradian interaction), and that it can be reversed by (ultradian) fasting (Figure 2C). In this sense, operant conditioning usually depends on food deprivation, so if our prediction is true then two studies only differing in the operant conditioning procedure can be contradictory because of this confounder. Indeed, many examples can be drawn even from our simplified model that demand to ponder temporal interactions among different scales and ranges when designing experiments or analyzing biological data.

Only by considering biological clocks as a complex interplay between complementary periodicities we shall understand the cyclical and temporal features of life on Earth and start correcting the fact that “the time is out of joint” (Hamlet, Act I, Scene V).

## Author Contributions

RL, PVA and DAG designed the study, analyzed and discussed the results and wrote the article. RL designed and ran the mathematical model.

## Conflict of Interest Statement

The authors declare that the research was conducted in the absence of any commercial or financial relationships that could be construed as a potential conflict of interest.
