# Supplementary material for: The Times of Our Lives: Interaction Among Different Biological Periodicities
Source: Front Integr Neurosci. 2018 Mar 13;12:10. doi: 10.3389/fnint.2018.00010 (PMC5859086; doi:10.3389/fnint.2018.00010)
Supplement: Supplementary file 1 [file Data_Sheet_1.pdf]

Laje R., Agostino P. V., and Golombek D. A.
The times of our lives: interaction among different biological periodicities

Supplementary Material

Supplementary Data: model equations

1. SCN oscillator

We chose to represent the SCN activity by an isolated harmonic oscillation, since we don't consider any feedback loop onto the SCN. The overall activity is represented by the SCN variable that oscillates between 0 and 1 with period *T*:

$$\frac{d\mathrm{SCN}}{dt} = y \tag{1}$$

$$\frac{dy}{dt} = -\left(\frac{2\pi}{T}\right)^2 (\mathrm{SCN} - 0.5) \tag{2}$$

Experimental manipulation: Normal Zeitgeber period is T=1440min; short Zeitgeber period is T=1100min (SH condition).

2. HPA axis

The HPA axis is modeled by an adapted Goodwin's model (Goodwin 1965, Gonze 2011) with parameter values giving an endogenous ultradian periodicity of 8-10 oscillations a day. CRH is released at a rate a_{A1} that is modulated by the circadian rhythm SCN and inhibited by CORT, while its concentration decreases with constant rate k_{A1}. ACTH is produced by CRH (a_{A2}) and decays with constant rate k_{A2}. CORT is produced by ACTH (a_{A3}) and is degraded at a constant rate k_{A3}:

$$\frac{d\mathrm{CRH}}{dt} = a_{A1}\mathrm{SCN}^4\,\mathrm{F}_A(\mathrm{CORT}) - k_{A1}\mathrm{CRH} \tag{3}$$

$$\frac{d\mathrm{ACTH}}{dt} = a_{A2}\mathrm{CRH} - k_{A2}\mathrm{ACTH} \tag{4}$$

$$\frac{d\mathrm{CORT}}{dt} = a_{A3}\mathrm{ACTH} - k_{A3}\mathrm{CORT} \tag{5}$$

The function F_A(CORT) simulates the inhibition of CRH production when CORT is sufficiently high, and is modeled by a decreasing sigmoid function:

$$\mathrm{F}_A(\mathrm{CORT}) = \frac{1}{1 + \exp(\beta_A(\mathrm{CORT} - \mathrm{CORT}_0))}$$

CORT_0 is the level of CORT at which the production of CRH is reduced by half.
Some limitations are worth considering, for instance CORT in this model doesn't display a higher morning peak (cortisol-awakening response; Postnova et al. 2013). Among the simplifications we can mention the absence of a negative feedback loop representing the known inhibition of ACTH by CORT.

3. HPT axis

Although the HPT axis has a much larger time scale than the HPA axis (circannual versus ultradian, respectively), their similar feedback loop architecture allows us to use a very similar model to represent HPT activity. The production of TRH is modulated by the SCN with rate a_{T1} and is inhibited by T3 and neuronal activity in the Arcuate (ARC) through sigmoid functions F_T(T3) and F_N(ARC). The degradation of TRH depends on itself as usual with constant rate k_{T1}. The production of TSH (rate a_{T2}) is modulated positively by TRH and negatively by EYA3, while its degradation has constant rate k_{T2}. Finally, TSH produces T3 at constant rate a_{T3} and T3 decays with constant rate k_{T3}:

$$\frac{d\mathrm{TRH}}{dt} = a_{T1}\mathrm{SCN}\,\mathrm{F}_T(\mathrm{T3})\,\mathrm{F}_N(\mathrm{ARC}) - k_{T1}\mathrm{TRH} \quad (6)$$

$$\frac{d\mathrm{TSH}}{dt} = a_{T2}\mathrm{TRH}\,\mathrm{EYA3}^4 - k_{T2}\mathrm{TSH} \quad (7)$$

$$\frac{d\mathrm{T3}}{dt} = a_{T3}\mathrm{TSH} - k_{T3}\mathrm{T3} \quad (8)$$

The negative feedback loop from T3 onto TRH is represented by the inhibition function F_T(T3):

$$\mathrm{F}_T(\mathrm{T3}) = \frac{1}{1+\exp(\beta_T(\mathrm{T3}-\mathrm{T3}_0))}$$

Inhibitory input to HPT from HNS is known to be mediated by direct projections from NPY-releasing ARC neurons onto TRH neurons in PVN (Sarkar and Lechan 2003). This is represented by the inhibition function F_N(ARC):

$$\mathrm{F}_N(\mathrm{ARC}) = \frac{1}{1+\exp(\beta_N(\mathrm{ARC}))}$$

Experimental manipulation: Fasting (or high NPY levels; Sarkar and Lechan 2003) is modeled as an increased efficacy of the ARC→HPT connection: β_N=0.1 for normal values (fed) and β_N=25 for high values (fasting).

EYA3 is modeled as a two-variable system tuned to display oscillations when driven by the SCN. For the sake of simplicity our choice was an adapted Fitzhugh-Nagumo model with auxiliary variable W1:

$$\tau_E\frac{d\mathrm{EYA3}}{dt} = \mathrm{EYA3} - \frac{1}{3}(\mathrm{EYA3}-2)^3 - \mathrm{W}_1 + b_E\mathrm{SCN}\,\mathrm{F}_E(\mathrm{W}_2) - 2 \quad (9)$$

$$\tau_E\frac{d\mathrm{W}_1}{dt} = \epsilon\,(\mathrm{EYA3} + c - d\mathrm{W}_1 - 2) \quad (10)$$

The inhibition of EYA3 expression by Melatonin is represented by the decreasing sigmoid function $F_E(W_2)$:

$$\mathrm{F}_E(\mathrm{W}_2) = \frac{0.1^2}{0.1^2 + {\mathrm{W}_2}^2}$$

Similarly, the equations for the evolution of Melatonin and its auxiliary variable W_2 are:

$$\tau_M\frac{d\mathrm{Mel}}{dt} = \mathrm{Mel} - \frac{1}{3}(\mathrm{Mel}-2.5)^3 - 1.5\mathrm{W}_2 + b_M\mathrm{PP} - 2.5 \quad (11)$$

$$\tau_M\frac{d\mathrm{W}_2}{dt} = \epsilon\,(\mathrm{Mel} + c - d\mathrm{W}_2 - 2.5) \quad (12)$$

Melatonin oscillations are driven by the photoperiod: PP=1 during the dark phase and zero otherwise.
Experimental manipulation: the light phase is defined as SCN>0.2 in long photoperiod (LP and SH conditions), and SCN>0.8 in short photoperiod (SP condition).

4. HNS axis

This is the most speculative part of the model, since little is known about the involved mechanisms and brain regions. We take as starting points the recent works by Prendergast and Zucker (2016) and Buijs et al. (2017), and the references therein. It is not known whether the activity in ARC is tonic or phasic (Prendergast and Zucker 2016), but striatal DA levels oscillate in synchrony with levels of locomotor activity (around 2.5 hours; Blum et al. 2014), so we assume it is phasic.
The model takes into account the proposed interactions among the arcuate nucleus (ARC), the substantia nigra / ventral tegmental area (VTA), and nucleus accumbens (Nac). Each region is modeled as a mean-field firing rate subpopulation with a Wilson-Cowan additive model (Hoppensteadt and Izhikevich 1997):

$$\tau_N \frac{d\mathrm{ARC}}{dt} = -\mathrm{ARC} + \mathrm{Q}\left(\rho_1(0.75 + (1 - \mathrm{SCN})) + \alpha_1 \mathrm{ARC} + \beta_1 \mathrm{NAc}\right) \quad (13)$$

$$\tau_N \frac{d\mathrm{VTA}}{dt} = -\mathrm{VTA} + \mathrm{Q}\left(\rho_2 + \alpha_2 \mathrm{VTA} - \beta_2 \mathrm{ARC}\right) \quad (14)$$

$$\tau_N \frac{d\mathrm{NAc}}{dt} = -\mathrm{NAc} + \mathrm{Q}\left(\rho_3 + \alpha_3 \mathrm{NAc} + \beta_3 \mathrm{VTA} - \gamma_3 \mathrm{ARC}\right) \quad (15)$$

The activity of every subpopulation decays with time constant τ_N and increases according to a sigmoid activation function Q whose argument is the algebraic sum of all the inputs to the subpopulation. Self-connections are all positive (α_1, α_2, α_3). The connection VTA→NAc is excitatory (Nicola et al. 1996), while the connection ARC→VTA is inhibitory (Narita et al. 2001) as the connection ARC→NAc (Prendergast and Zucker 2016, van den Heuvel et el. 2014). We assume that the connection NAc→ARC is excitatory. For the sake of simplicity we dropped the known influence of ARC out of the HNS onto SCN. The increasing sigmoid activation function Q is:

$$\mathrm{Q}(x) = \frac{1}{1 + \exp(-x)}$$

Experimental manipulation: Higher levels of Dopamine lead to larger oscillation periods in HNS (Blum et al. 2014). We modeled this by increasing the HNS-global time constant: τ_N=28min for low (normal) DA levels, and τ_N=118min for intermediate DA levels. Even larger values of τ_N lead to a T3 profile (Figure 2C) that is indistinguishable from the "Fed, normal DA" condition (data not shown).

References:

- Buijs, F.N., Guzmán-Ruiz, M., León-Mercado, L., Basualdo, M.C., Escobar, C., Kalsbeek, A., Buijs, R.M. (2017). Suprachiasmatic Nucleus Interaction with the Arcuate Nucleus; Essential for Organizing Physiological Rhythms. *eNeuro* **4**(2). pii: ENEURO.0028-17.2017. doi: 10.1523/ENEURO.0028-17.2017.
- Narita, M., Funada, M., Suzuki, T. (2001). Regulations of opioid dependence by opioid receptor types. *Pharmacology & Therapeutics*, **89**(1):1-15, doi: 10.1016/S0163-7258(00)00099-1.
- Nicola, S.M., Kombian, S.B., Malenka, R.C. (1996). Psychostimulants depress excitatory synaptic transmission in the nucleus accumbens via presynaptic D1-like dopamine receptors. *Journal of Neuroscience* **16**(5):1591-604.
- Prendergast, B.J., Zucker, I. (2016). Ultradian rhythms in mammalian physiology and behavior. *Curr. Opin. Neurobiol.* **40**:150-154. doi: 10.1016/j.conb.2016.07.011.

- van den Heuvel JK, Furman K, Gumbs MC, Eggels L, Opland DM, Land BB, Kolk SM, Narayanan NS, Fliers E, Kalsbeek A, DiLeone R.J., la Fleur S.E. (2015). Neuropeptide Y activity in the nucleus accumbens modulates feeding behavior and neuronal activity. *Biol Psychiatry* **77**:633-641.
- Sarkar, S., Lechan, R.M. (2003). Central Administration of Neuropeptide Y Reduces α-Melanocyte-Stimulating Hormone-Induced Cyclic Adenosine 5′-Monophosphate Response Element Binding Protein (CREB) Phosphorylation in Pro-Thyrotropin-Releasing Hormone Neurons and Increases CREB Phosphorylation in Corticotropin-Releasing Hormone Neurons in the Hypothalamic Paraventricular Nucleus. *Endocrinology*, **144**(1):281–291, doi: 10.1210/en.2002-220675

5. Simulations of the full model

In this work all simulations are performed by numerically integrating all equations at the same time, and using a fixed set of parameter values as shown below. As very different timescales are builtin in the system (ranging from about 1 hour in HPA to 1 year in HPT), numerical integration was performed with MATLAB's variable-step ode45 solver for stiff equations.
MATLAB code is available as part of the Supplementary Material.

Table1: Parameter values. All parameter values are presented as nondimensional quantities.

**SCN system**		
T	1440 min (SP, LP)<br>1100 min (SH)	*Experimental manipulation
photoperiod	0.2 (SP)<br>0.8 (LP, SH)	*Experimental manipulation
**HPA axis**		
a_{A1}	0.063	
a_{A2}	21	
a_{A3}	210	
k_{A1}	0.03639	
k_{A2}	0.09704	
k_{A3}	0.02079	
β_A	0.1	
CORT_0	150	
**HPT axis**		
a_{T1}	0.0000117	
a_{T2}	0.000039	
a_{T3}	0.039	
k_{T1}	0.000006758	
k_{T2}	0.000018022	
k_{T3}	0.000003862	
β_T	0.1	
$\mathrm{T3}_0$	150	
τ_E	33.3333	
τ_M	66.6666	
ϵ	0.08	
b_E	0.75	
b_M	1	
c	0.7	
d	0.8	

**HNS axis**		
τ_N	28 (DA low)<br>118 (DA med)	*Experimental manipulation
β_N	0.1 (NPY low = normal fed)<br>25 (NPY high = fasting)	*Experimental manipulation
α_1	2	
α_2	10	
α_3	2	
β_1	8	
β_2	10	
β_3	8	
γ_3	2	
ρ_1	-6	
ρ_2	0	
ρ_3	-6	

Supplementary Figures: model numerical simulations

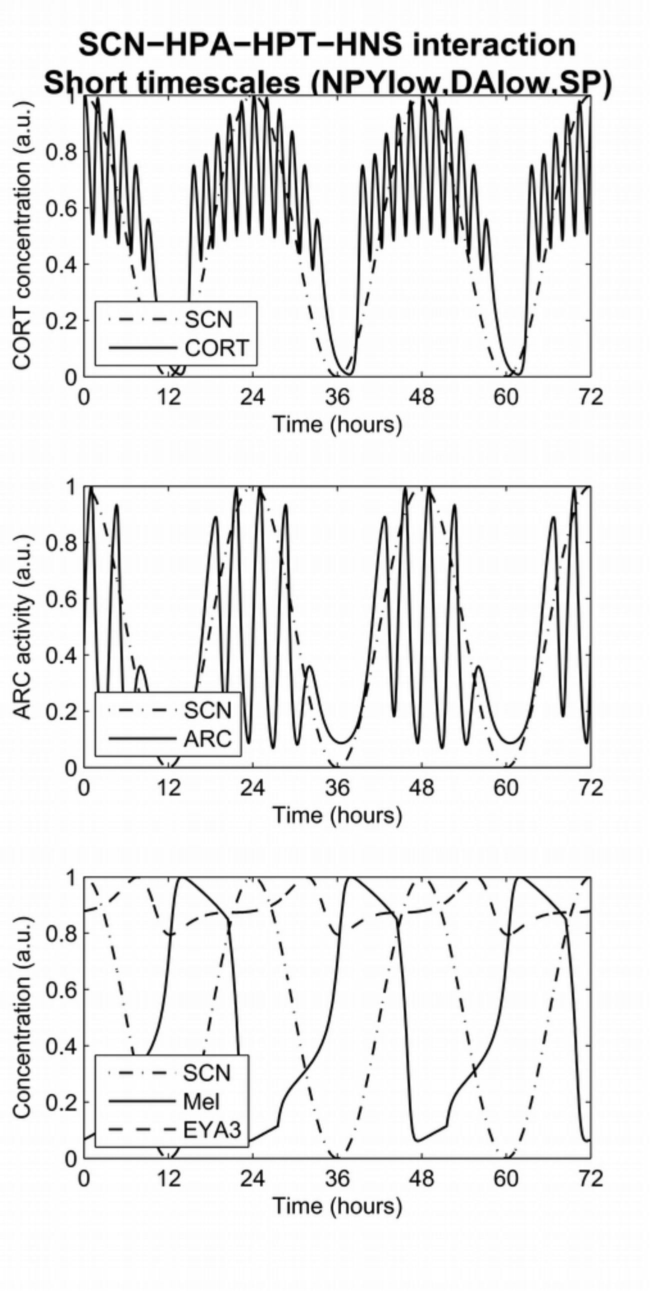

Figure S1. Behavior of the model at short timescales.
Top: CORT concentration displaying ultradian oscillations. The model doesn't take into account the known cortisol-awakening response (a higher Cortisol morning peak).
Middle: ARC firing rate. It is assumed to be phasic rather than tonic (Prendergast and Zucker 2014).
Bottom: Melatonin and EYA3 interaction at short photoperiod (SP). EYA3 doesn't display a high daily peak because of inhibition from Mel. See Figure S2.

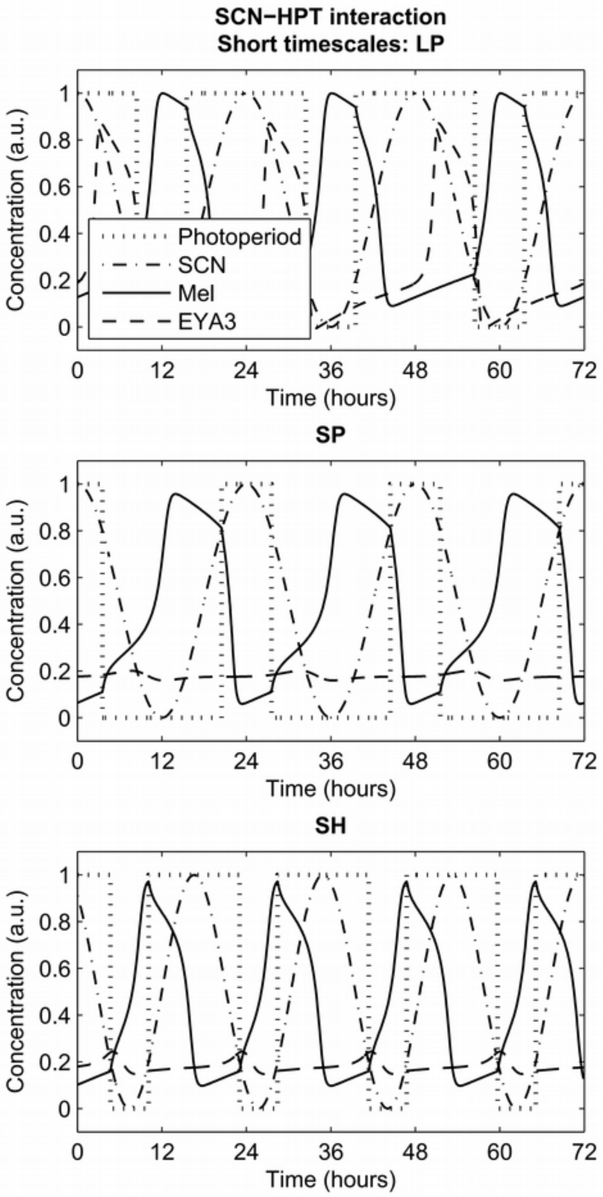

Figure S2. Short timescales of first experimental manipulation (see Figure 2B in the main text).
Top: At long photoperiod (LP), EYA3 displays a high peak because Mel is out of phase with it.
Middle: At short-photoperiod (SP), a broader Mel peak inhibits EYA3 expression.
Bottom: Prediction of the model at long photoperiod in a shorter Zeitgeber period. The daily Mel peak again inhibits EYA3 expression.
